# Overexpression of TICRR and PPIF confer poor prognosis in endometrial cancer identified by gene co-expression network analysis

**DOI:** 10.18632/aging.202417

**Published:** 2021-01-20

**Authors:** Linlin Yang, Yunxia Cui, Xiao Sun, Yudong Wang

**Affiliations:** 1 Department of Gynecologic Oncology, The International Peace Maternity and Child Health Hospital, School of Medicine, Shanghai Jiao Tong University, Shanghai, China; 2 Shanghai Municipal Key Clinical Specialty, Shanghai, China; 3 Shanghai Key Laboratory of Embryo Original Disease, Shanghai, China

**Keywords:** bioinformatics analysis, *in vitro* experiment, WGCNA, endometrial carcinoma, prognosis

## Abstract

The incidence of endometrial cancer (EC) is intensively increasing. However, due to the complexity and heterogeneity of EC, the molecular targeted therapy is still limited. The reliable and accurate biomarkers for tumor progression are urgently demanded. After normalizing the data from Gene Expression Omnibus (GEO) and The Cancer Genome Atlas (TCGA), we utilized limma and WGCNA packages to identify differentially expressed genes (DEGs). The copy number variations of candidate genes were investigated by cBioPortal. Enrichment pathways analysis was performed by ClueGO and CluePedia. The methylation status was explored by UALCAN. ROC curve and survival analysis were conducted by SPSS and Kaplan–Meier. Infiltration immune cells in microenvironment were analyzed by TISIDB. Gene Set Enrichment Analysis (GSEA) and Gene Set Variation Analysis (GSVA) were applied to explore potential biological pathways. Immunohistochemistry staining (IHC), cell proliferation, cell apoptosis, colony formation, migration, invasion and scratch-wound assays were performed to investigate the function of key genes *in vitro*. In this study, four expression profile datasets were integrated to identify candidate genes. Combined with WGCNA analysis, the top ten candidates were screened out, whose abnormal methylation patterns were extremely correlated with their expression level and they were associated with tumor grades and predicted poor survival. GSEA and GSVA demonstrated they were involved in DNA replication and cell cycle transition in EC. Gene silencing of TICRR and PPIF dramatically inhibited cell growth, migration and epithelial-mesenchymal transition (EMT) and enhanced progesterone sensitivity. Additionally, from DrugBank database, cyclosporine may be effective for PPIF targeted therapy. By integrative bioinformatics analysis and *in vitro* experiments, our study shed novel light on the molecular mechanisms of EC. TICRR and PPIF may promise to be potential therapeutic targets for endometrial cancer.

## INTRODUCTION

Since the morbidity and mortality of endometrial cancer (EC) is constantly increasing per year [1, 2], EC has become the fourth most common malignancy in the female reproductive system. There are around 170,000 diagnosed cases and 36,000 deaths of EC annually [3], which accounts for approximately 5.7% of new cases and 3% of deaths in all types of tumors [4]. The 5-year disease-specific survival of patients diagnosed in the early stages is 74.2-90.8%. For women in progressive stage III or stage IV, it is 57.3-66.2% and 20.1-25.5% respectively [5], while for patients with recurrent or metastatic disease, it remains as low as 16% [6]. Although substantial efforts have been made to explore the mechanisms of carcinogenesis [7–10], owing to the complexity and heterogeneity of EC, the etiology of EC still remains obscure and the progress in targeted or personalized therapy is limited. Therefore, the exploration of targeted molecules and explanation of tumor mechanisms are indispensable.

With the advances in bioinformatics technology, microarray and high-throughput sequencing platform provided new perspectives for cancer study [11]. In order to avoid differential gene research bias of small sample sizes in individual studies, this current research performed a comprehensive and systematic bioinformatics analysis of EC. Besides, weighted gene co-expression network analysis (WGCNA) as a novel and effective biology method [12], which illustrates gene expression data through constructing gene networks based on similarities in expression pattern among samples, was adopted to identify the most connected genes that were associated with clinical features [13]. After overlapping the genes in differential expression and co-expression network, the common genes were found. The Cancer Genome Atlas (TCGA) was used to analyze somatic mutation status of hub genes, through which we could find the distributions of mutation frequencies, types and contexts in EC [14]. The interaction between tumor and immune defense system plays a critical role in cancer progression and pharmacotherapy efficacy. To investigate associations between hub genes and immune factors, such as mature lymphocytes, immunomodulators and chemical chemokines, the web portal of TISIDB was applied [15]. MEXPRESS and UALCAN analysis were performed to explore epigenetic regulation of gene especially the methylation status in promoter region and their correlation with gene expression [16]. Gene Set Enrichment Analysis (GSEA) and Gene Set Variation Analysis (GSVA) were utilized to demonstrate underlying biological functions of hub genes [17].

In this study, we implemented an integrated bioinformatics analysis of EC using GSE17025, GSE63678 and GSE115810 and TCGA UCEC databases. In combination with WGCNA strategy, the most remarkable genes were identified. Then, the somatic mutation status, methylation patterns, gene expression profiles and survival analysis were performed by cBioPortal, UALCAN, Gene Expression Profiling Interactive Analysis (GEPIA), Human Pathology Atlas (HPA) and Kaplan-Meier method, respectively. Moreover, GSEA and GSVA analysis were applied to verify the most involved pathways of the real hub genes. Finally, two DEGs (TICRR and PPIF) were generated, which may become potential prognostic indicators and therapeutic targets for endometrial cancer.

## RESULTS

### Identification of robust DEGs between endometrial carcinoma and normal tissues

**Figure 1:**
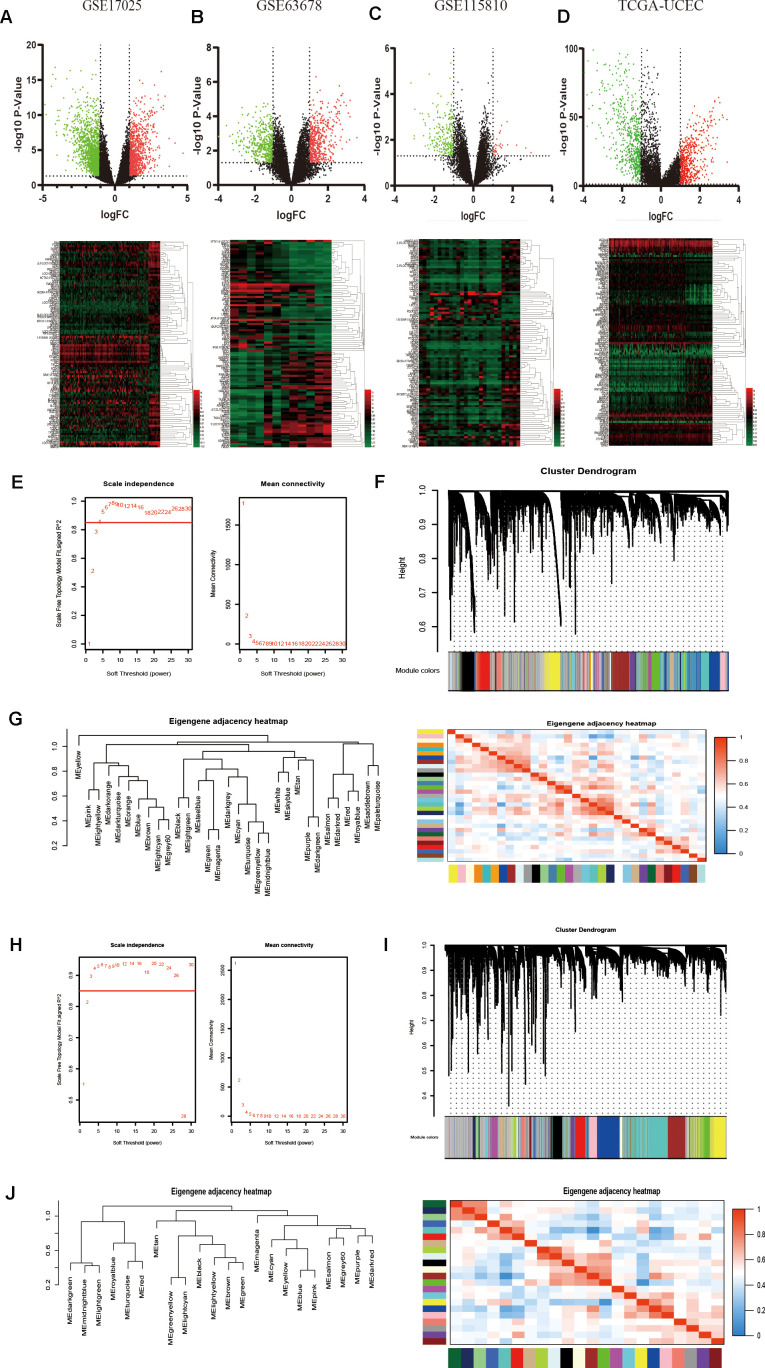
**Identification of DEGs among GEO and TCGA-UCEC datasets and construction of co-expression modules by WGCNA package.** (**A**–**D**) The volcano plots and heatmaps of DEGs in GSE17025, GSE63678, GSE115810 and TCGA-UCEC databases, respectively. In the heatmap, red indicates relative upregulation of gene expression; black indicates no significant change; green indicates downregulation of gene expression. (**E**) Analysis of the scale-free fit index and mean connectivity for various soft-thresholding powers for TCGA samples. (**F**) Dendrogram of differentially genes clustered based on a dissimilarity measure (1-TOM) for TCGA samples. (**G**) Dendrogram of consensus module eigengenes and heatmap of the adjacencies for TCGA. (**H**) Analysis of network topology for various soft-threshold powers for GSE17025 data. (**I**) Clustering dendrogram of genes in GSE17025 with dissimilarity based on topological overlap, together with assigned module colors. (**J**) Visualizing the gene network using a heatmap plot of GSE17025 data.

Three gene expression datasets about EC from the GEO database, including GSE17025, GSE63678 and GSE115810, and TCGA UCEC RNA-sequencing data were used to identify DEGs in EC (Table 1). After data preprocessing and standardization, the limma and DESeq2 R packages were utilized to screen out DEGs [18]. On the basis of the threshold of P value < 0.05 and |log fold change (FC)| > 2, DEGs were obtained and differential results were shown in the volcano plots, respectively. The expression profiles of the top 100 DEGs were acquired from the four datasets and visualized by cluster heatmaps, as shown in Figure 1A–1D, respectively.

**Table 1:** Details of the datasets.

**Dataset**	**sample**	**normal**	**tumor**	**platform**	**reference**
GSE17025	endometrium	12	91	GPL570	Day et al.(2011)
GSE63678	endometrium	5	7	GPL571	Pappa et al.(2015)
GSE115810	endometrium	3	24	GPL96	Hermyt et al.(2019)
TCGA UCEC	endometrium	35	333	-	-

### Construction of weighted gene co-expression network and identification of the core modules

**Figure 2:**
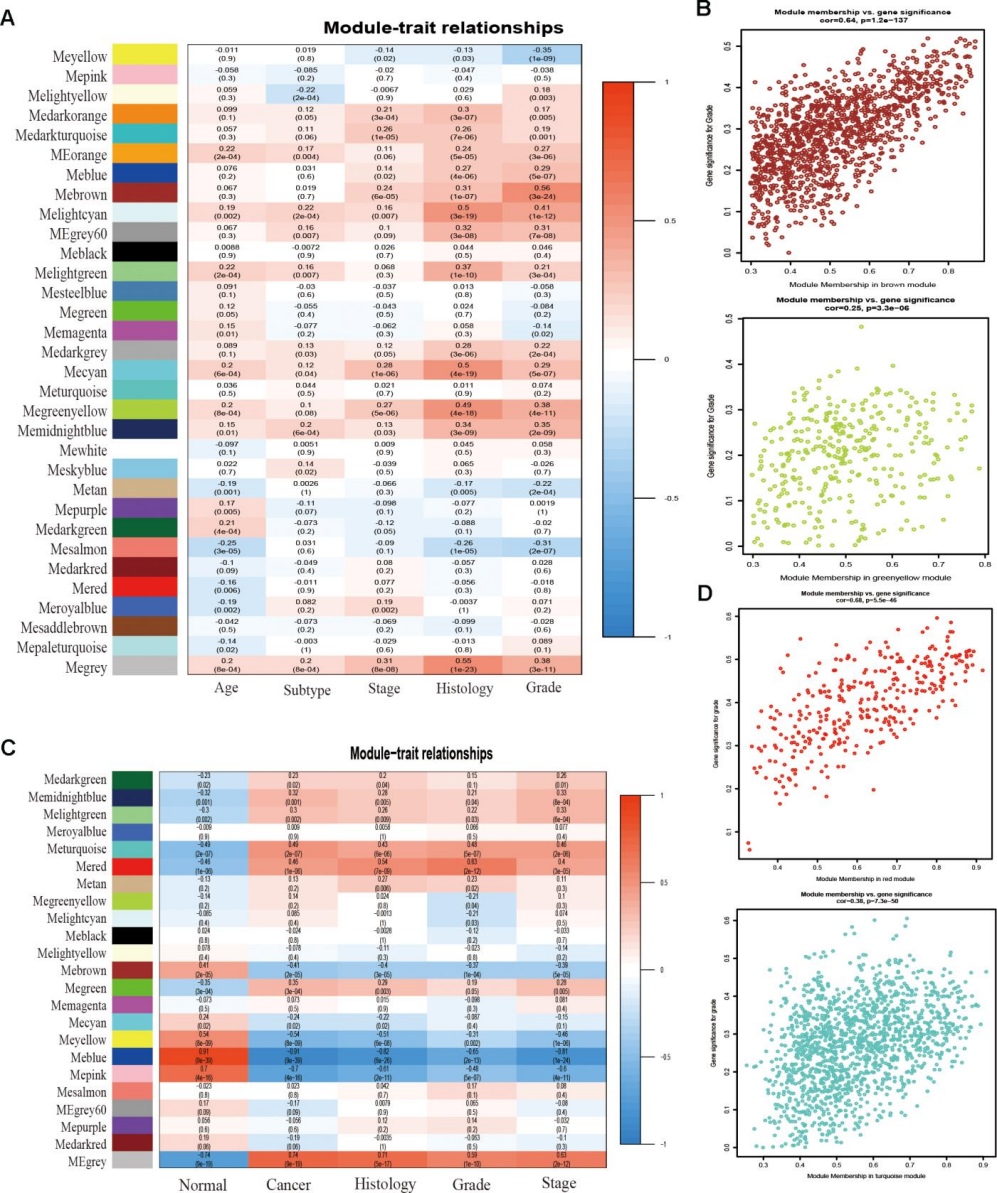
**Correlation between module eigengenes and clinical traits.** (**A**) Correlation between modules and traits of TCGA data. (**B**) Scatter plots of GS versus the MM in the brown and green-yellow modules of TCGA data. (**C**) Correlation between modules and traits of GSE17025 data. (**D**) Scatter plots of module eigengenes in turquoise and red modules of GSE17025.

To detect the key modules most relevant to EC clinical traits, WGCNA was performed on the four datasets, respectively. The data of EC were acquired from TCGA database. After data standardization and preprocessing, the 10 000 most significant genes based on expression values of EC were applied to set up the weight co-expression network. After filtering out the aberrant samples, the soft threshold power value β was defined as four to build the adjacency matrix by the power function (scale free R^2^ = 0.89) (Figure 1E), The co-expression modules were performed by hierarchical clustering and dynamic branch cutting (Figure 1F). The dendrogram and heatmap of samples (Figure 1G) were utilized to screen out groups of relevant eigengenes. From the heatmap of module–trait correlations (Figure 2A), we explored that the brown and green-yellow modules were the most highly correlated with clinical features. Moreover, the scatterplots of gene significance (GS) vs module membership (MM) in the two selected modules were plotted respectively (Figure 2B). In addition, sample cluster of GSE17025 was performed to detect data quality in the microarray and to remove outlier samples. We set the soft threshold as three (Figure 1H) and twenty-two modules were identified (Figure 1I). Besides, we clustered eigengenes on the basis of their correlation and equivalent results were demonstrated by the heatmap (Figure 1J). The ME in the turquoise and red module illustrated a high correlation with tumor status, respectively (Figure 2C, 2D) illustrated the correlation between MM and GS in the two modules, respectively. Meanwhile, the cluster analysis images for GSE115810 and GSE63678 were shown in Supplementary Figures 1, 2, the black module in GSE115810 and the pink module in GSE63678 tended to be significantly correlated with tumor traits among other modules.

### Identification of hub genes

**Figure 3:**
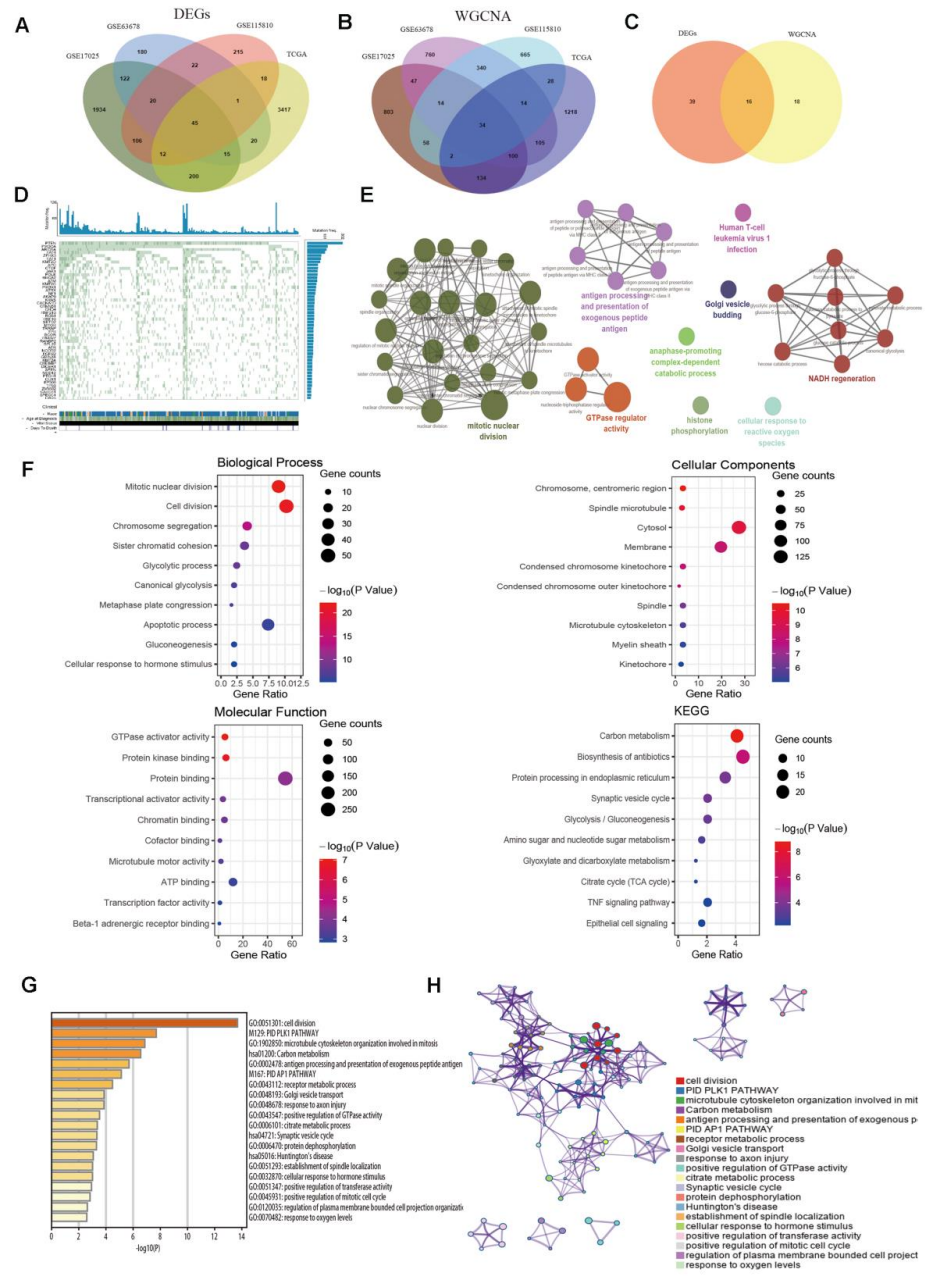
**Identification of common hub genes and functional enrichment analysis.** (**A**) Venn diagram shows the intersection genes of GSE17025, GSE63678, GSE115810 and TCGA-UCEC. (**B**) Essential genes were found among the selected modules through co-expression network. (**C**) The intersection common genes of essential genes and DEGs. (**D**) Top 50 somatic mutated genes in EC. (**E**) The functional annotation analysis of hub genes was performed by ClueGO and CluePedia. (**F**) Biological Process (BP) terms, Cellular Component (CC) terms, Molecular Function (MF) terms and KEGG analysis for genes. (**G**) Boxplot of enriched terms across input gene lists by Metascape, colored by P-values. (**H**) Network of enriched terms by Metascape.

Venn diagram was performed to identify common genes among the four aforementioned datasets. As demonstrated in Figure 3A, 45 common DEGs were detected (Supplementary Table 1). The selected modules that correlated with tumor traits through co-expression network were also overlapped (Figure 3B and Supplementary Table 2). Subsequently, a total of sixteen key genes were screened out from both DEG network and co-expression network (Figure 3C and Supplementary Table 3). The genes were shown in Table 2. In order to explore whether the key genes showed somatic mutations in EC, we detected mutations in tumor-related genes and found PTEN (71.46%), PIK3CA (50.6%) and ARID1A (40.77%) were most frequently mutated genes (Figure 3D). Next, the biological function of the common genes and their co-expression genes was analyzed using ClueGO and CluePedia, showing enrichment in mitotic nuclear division, GTPase regulator activity and NADH regeneration pathway (Figure 3E). Then we conducted GO and KEGG analyses (Figure 3F), which indicated that genes were notably involved in mitotic nuclear division, carbon metabolism and TNF signaling pathway (Table 2). We analyzed the results in Metascape database (Figure 3G, 3H), showing similar enrichment in cell division, receptor metabolic process and positive regulation of GTPase activity, which were consistent with our findings.

**Table 2:** List of sixteen hub genes identified by venn analysis.

**Gene symbol**	**Description**
UBE2C	ubiquitin conjugating enzyme E2 C
SOWAHC	sosondowah ankyrin repeat domain family member C
CYR61	cellular communication network factor 1
MYO15B	myosin XVB
ARHGAP10	Rho GTPase activating protein 10
GNG11	G protein subunit gamma 11
LYPLA2	lysophospholipase 2
PLS1	plastin 1
PPIF	peptidylprolyl isomerase F
BIRC5	baculoviral IAP repeat containing 5
PRRC2A	proline rich coiled-coil 2A
TICRR	TOPBP1 interacting checkpoint and replication regulator
MUC1	mucin 1, cell surface associated
VAMP8	vesicle associated membrane protein 8
WT1	WT1 transcription factor
CXCL12	C-X-C motif chemokine ligand 12

### Real hub genes validation in the TCGA dataset

**Figure 4:**
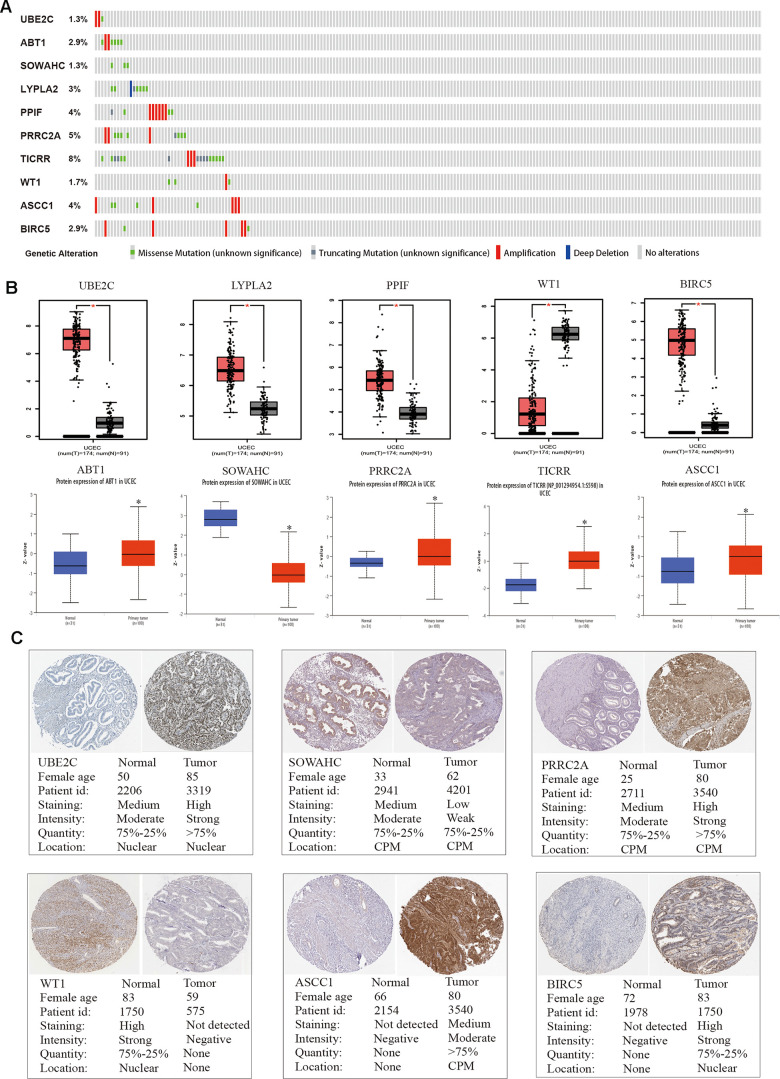
**Genetic alterations and protein expression of hub genes in TCGA.** (**A**) A visual summary on a query of genetic alteration of 10 hub genes in TCGA dataset. (**B**) Transcriptional level of each gene was identified in both GEPIA and UALCAN database. *p < 0.05 compared with normal endometrial tissues. (**C**) Validation of hub genes by The Human Protein Atlas database.

We adopted the top ten variant genes that had not been researched in EC for subsequent analysis. OncoPrint of CBioPortal was utilized to demonstrate the ten hub genes’ alteration pattern in TCGA EC patients and the alteration frequency of each common gene was illustrated in Figure 4A, which was less than 8%. Then we applied GEPIA and UALCAN databases to explore the difference in expression level among tumor and normal tissues (Figure 4B). Results suggested that all these ten hub genes were abnormally differentially expressed in the tissues of endometrial cancer. Furthermore, immunohistochemistry (IHC) staining extracted from Human Protein Atlas database (HPA) also demonstrated the differential protein expression level of core genes, as shown in Figure 4C, which were in accordance with the transcriptional level. Above results confirmed that the hub genes we identified were reliable.

### Methylation status, survival analysis and efficacy evaluation of hub genes

**Figure 5:**
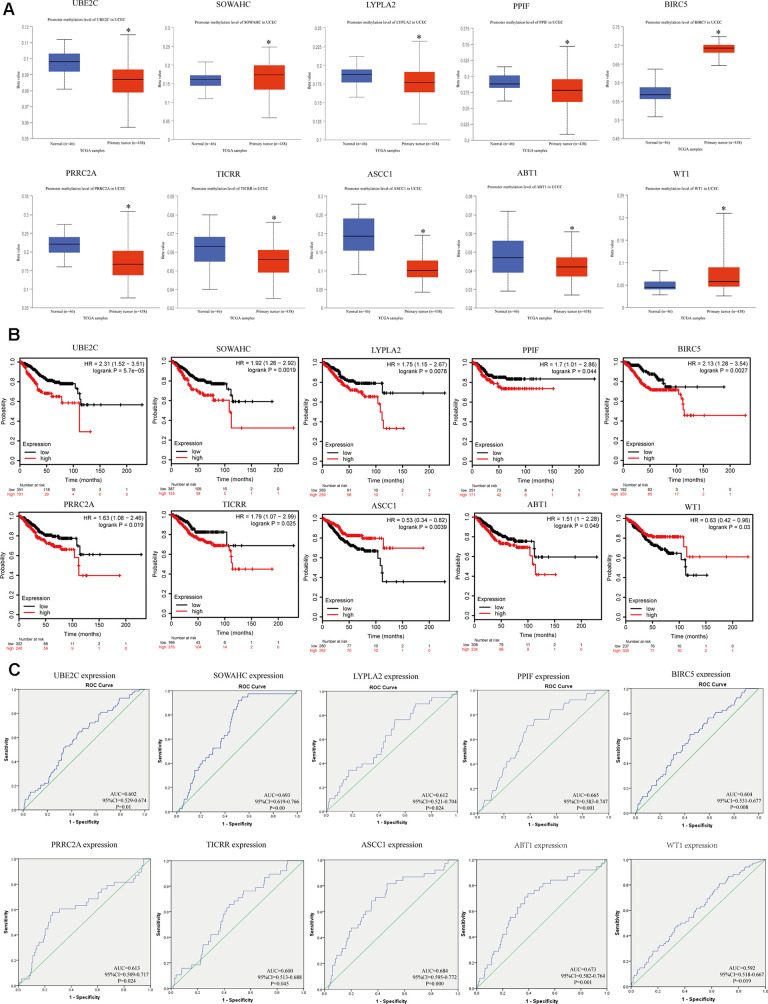
**Methylation status and survival analysis of the hub genes.** (**A**) Gene methylation status of ten hub genes were validated in TCGA database. *p < 0.05 compared with normal endometrial tissues. (**B**) Univariate survival analysis of the hub genes using the Kaplan-Meier curve. (**C**) Receiver operating characteristic (ROC) curve analysis and area under the curve (AUC) statistics was applied to evaluate the capacity of real hub genes to predict overall survival of patients.

We explored the methylation status of core genes to clarify potential mechanisms of their aberrant expression in EC tissues (Figure 5A). Results showed that they were all aberrantly methylated, which were extremely correlated with their expression level. Next, overall survival data was analyzed based on the low or high expression of each gene, which showed statistically significant difference (Figure 5B). Furthermore, ROC curve assessment was carried out to detect the capacity of real core genes to predict overall survival of patients by using SPSS and AUC values of ten genes were greater than 0.6 (Figure 5C), showing that they could be utilized as effective indicators to monitor prognosis.

### Verification the expression and function of TICRR and PPIF genes through *in vitro* experiments

**Figure 6:**
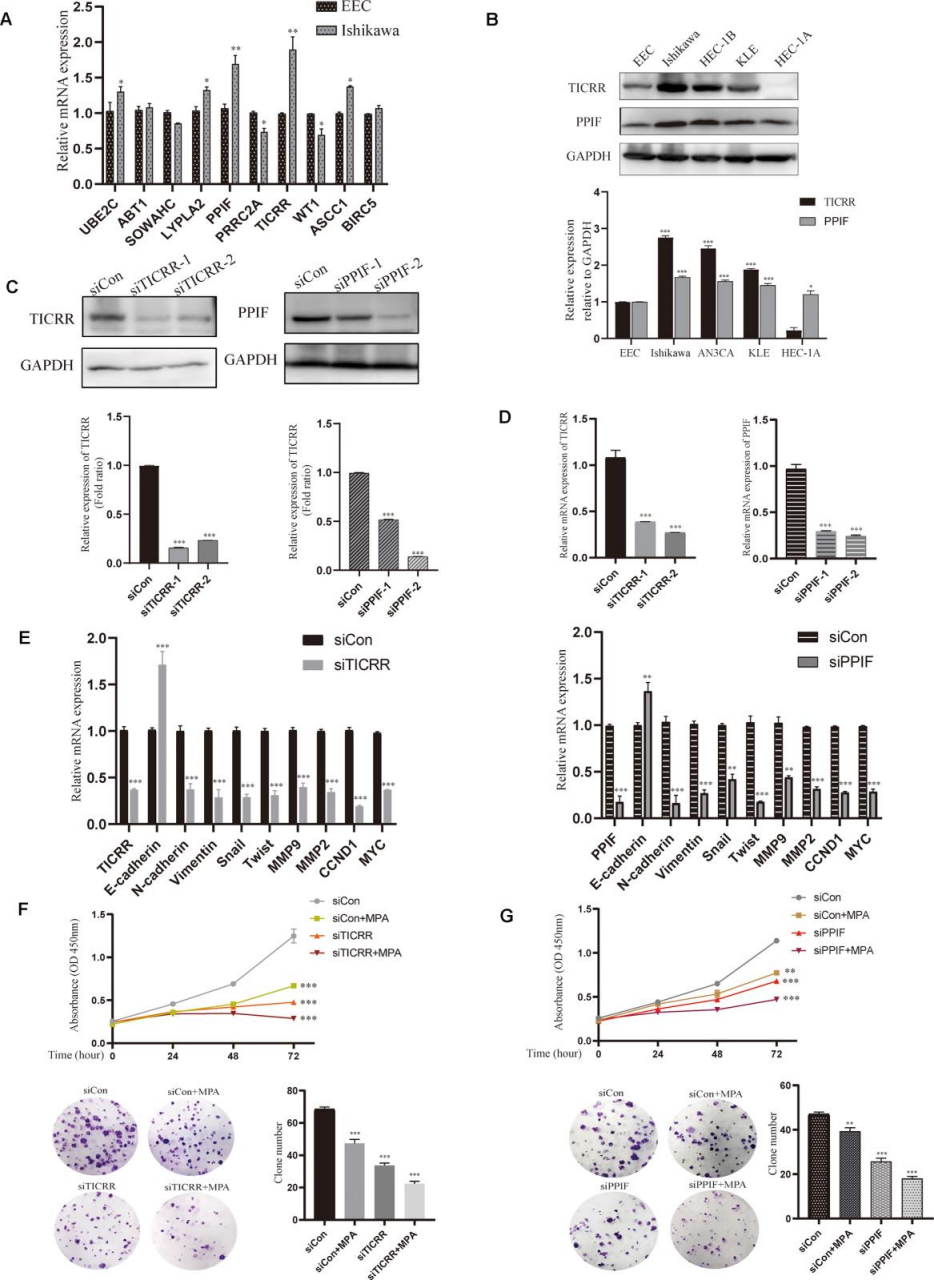
**Validation of hub genes by cell experiments *in vitro*.** (**A**) Identification of differentially expressed genes by qPCR assay in both normal endometrial epithelial cells and endometrial cancer cell lines. (**B**) Western blot assay showing the expression of TICRR and PPIF in EC cell lines and normal endometrial epithelial cells. (**C**) The effect of different siRNA on TICRR and PPIF gene silence by western blotting respectively. (**D**) TICRR and PPIF expression were examined by RT-PCR after transfected with siRNA for 24h. (**E**) Relevant molecular targets were verified by qRCR after transfection of siTICRR and siPPIF for 24hrs respectively. (**F**) Cell growth and clone formation of siTICRR transfected cells with or without MPA. (**G**) Cell growth and clone formation of siPPIF transfected cells with or without MPA. Data were shown as mean ± SD; *p < 0.05; **p < 0.01; ***p < 0.001.

**Figure 7:**
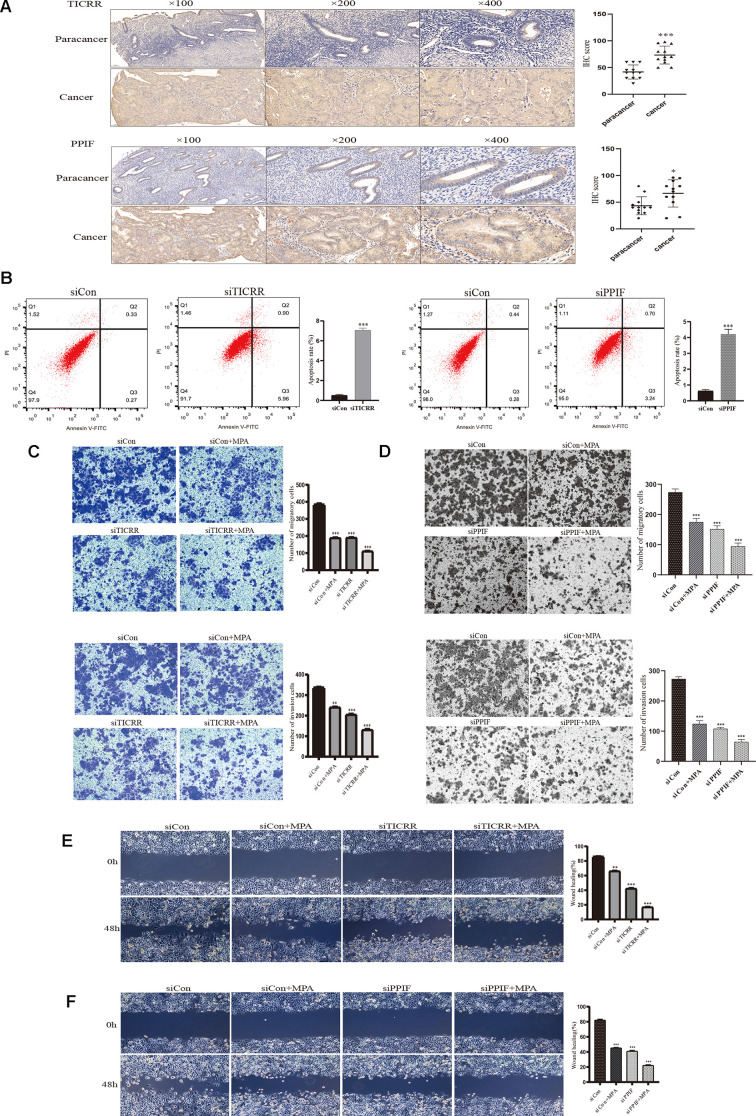
**IHC staining and functional verification of TICRR and PPIF genes.** (**A**), The expression of TICRR and PPIF in endometrial cancer and paracancer samples were assessed using IHC staining and the results of statistical analysis. (**B**) Analysis of apoptosis by Flow cytometry after transfection with siTICRR and siPPIF respectively. (**C**) Hec-1B-siTICRR cells were subjected to migration and invasion assays in the presence or absence of MPA. (**D**) Migration and invasion ability of siPPIF transfected cells in the presence or absence of MPA. (**E**) Wound-healing assays for Hec-1B-siTICRR cells. (**F**) Wound-healing assays for Hec-1B-siPPIF cells. Data were shown as mean ± SD; *p < 0.05; **p < 0.01; ***p < 0.001.

To evaluate the expression of differential genes at the cellular level, we performed qRT-PCR assays in both normal and endometrial cancer cells. Genes TICRR and PPIF were significantly overexpressed in cancer cells than in normal endometrial epithelial cells (EEC) compared with other genes, suggesting they may play a pivotal role in oncogenesis and were selected for the following research (Figure 6A). We then evaluated the expression patterns of TICRR and PPIF in a panel of endometrial cancer cell lines. Higher levels of TICRR and PPIF were observed in Ishikawa, HEC-1B and KLE cell lines compared to EEC cells (Figure 6B). These data indicated that TICRR and PPIF may be associated with endometrial cancer. Meanwhile, we knocked down the expression of TICRR and PPIF in the cancer cell lines by transfection of siTICRR and siPPIF respectively, siTICRR-1 and siPPIF-2 with high transfection efficiency were used for subsequent experiments (Figure 6C, 6D). Silencing of TICRR and PPIF led to enhance the expression of epithelial marker E-cadherin and decrease the mesenchymal marker N-cadherin and metastasis associated genes MMP9 as well as proliferation-related marker CCND1, suggesting their involvement in the progression of endometrial cancer (Figure 6E). Medroxyprogesterone acetate (MPA) as a conservative therapy is commonly applied to the early stage of endometrial carcinoma [19]. The knockdown of TICRR resulted in a potent decrease of cell growth and clone formation and enhanced the inhibitory effect of MPA (Figure 6F) and the downregulated PPIF yielded the similar results (Figure 6G). Meanwhile, we found that TICRR and PPIF positive staining were present at a higher level in tissues with endometrial cancer than in tissues of paracancer endometrium (Figure 7A). Next, siRNA knockdown of TICRR and PPIF respectively increased the percentage of apoptotic cells in Ishikawa cells (Figure 7B). Further, silencing of TICRR and PPIF respectively in Hec-1B cells significantly inhibited cell migration and invasion compared to control cells and elevated the effectiveness of progesterone (Figure 7C, 7D). The effect of TICRR on cell migratory capacity was also analyzed by wound-healing assay, revealing an apparent suppression of the migratory ability of Hec-1B cells transfected with TICRR siRNA in combination with MPA (Figure 7E). Moreover, the wound closure was reduced obviously in siPPIF-treated cells as well, making progesterone treatment more effective (Figure 7F).

### Association of hub genes expression with clinicopathologic parameters and immune infiltration

**Figure 8:**
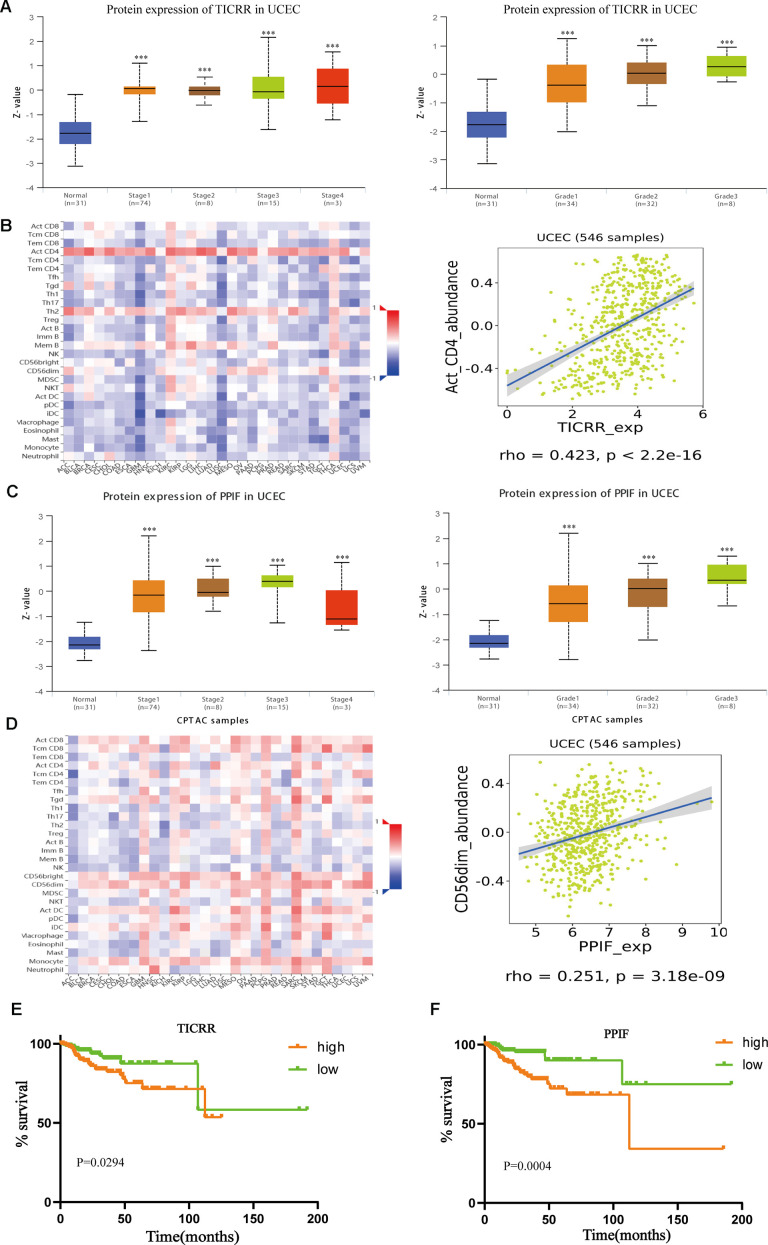
**Further exploration of the expression of TICRR and PPIF.** (**A**), Transcriptional expression of TICRR was significantly correlated with clinical stages and pathological grades of EC. ***p<0.001 compared with normal tissues. (**B**) Analysis of the correlation between the expression of TICRR and infiltrating immune cells (**C**), Correlation between PPIF and clinical stages and grades, respectively. ***p<0.001 compared with normal tissues. (**D**) Correlation between PPIF and immune cell populations. (**E**) Prognostic value of TICRR calculated by Kaplan-Meier analysis. (**F**) Overall survival analyzed using the Kaplan–Meier method with log-rank testing according to PPIF expression.

Further, we verified the expression of core genes in different pathological status. As illustrated in Figure 8A, mRNA expression of TICRR in EC samples were dramatically correlated with clinical stages and pathological grades. Similarly, upregulated expression of PPIF was notably correlated with the advanced pathological parameters in TCGA cohort (Figure 8C). Infiltrating immune cells were main components of tumor microenvironment and may play a vital role in tumor progression. Through the method of Microenvironment Cell Populations-counter [20], we explored the association between TICRR and immune cell populations. Results showed that TICRR had a correlation with activated CD4 T cell (Figure 8B), while there was weak correlation between PPIF and CD56dim natural killer cell in UCEC (Figure 8D). In addition, survival information and ROC curves of the two genes were also obtained using TCGA data, suggesting that high TICRR and PPIF expression tended towards poor prognosis of EC patients (Figure 8E, 8F), which were consistent with our former findings.

### GSEA and GSVA analysis of core genes

**Figure 9:**
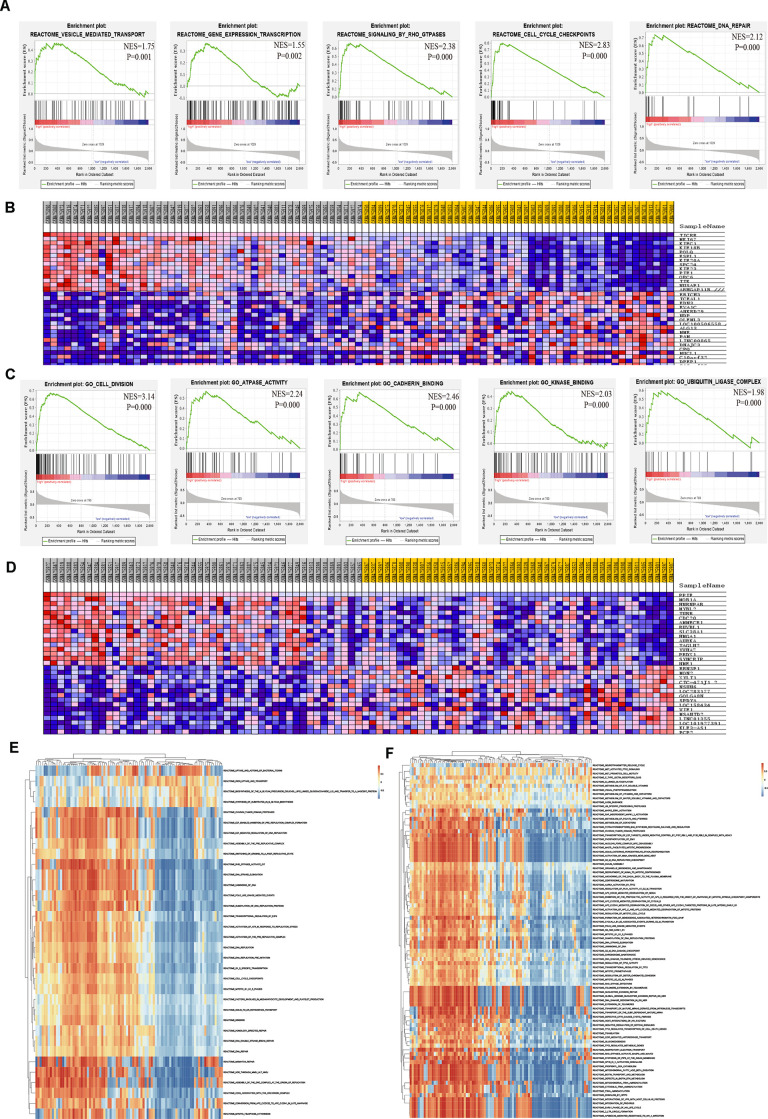
**Significant TICRR and PPIF-related genes and pathways in EC obtained by GSEA and GSVA.** (**A**) The most involved pathways in high-expression group of TICRR obtained by GSEA analysis. (**B**) Transcriptional expression profiles of the significant genes were shown in a heat map. (**C**) The most common functional gene sets in high-expression group of PPIF by GSEA. (**D**) Transcriptional expression profiles of the significant genes related with PPIF were shown in a heat map. (**E**, **F**) GSVA-derived clustering heatmaps of differential pathways for TICRR and PPIF, respectively.

To thoroughly investigate the potential biological functions of TICRR and PPIF in EC, GSEA and GSVA were applied to perform pathway analysis. Results of GSEA showed that the most involved pathways of TICRR were vesicle mediated transport, gene expression transcription, RHO GTPases signaling, cell cycle checkpoints and DNA repair pathway (Figure 9A). The top 50 significant genes were acquired by GSEA and shown in a heat map (Figure 9B). Meanwhile, GSEA enrichment analysis of PPIF indicated that cell division, ATPase activity, cadherin binding, kinase binding and ubiquitin ligase complex were the most significant pathways (Figure 9C) and the heat map of transcriptional expression profiles of 50 significant genes was shown in Figure 9D. Furthermore, GSVA analysis of TICRR confirmed that E2F mediated regulation of DNA replication, RHO GTPases activity and mitotic G1/S phrase pathway were remarkably statistically significant in the high-expression groups of TICRR (Figure 9E) and MAPK3/ERK1 activation and regulation of PLK1 at G2/M transition pathway were activated in high-expression groups of PPIF (Figure 9F), suggesting their involvement in the cell cycle transition and proliferative processes in progression of endometrial cancer. In addition, DrugBank database was used to explore drugs targeting hub genes (Supplementary Figure 3). Results showed that cyclosporine and triglyme were small molecule drugs targeting PPIF, which provided support for drug selection of PPIF targeted therapy.

## DISCUSSION

Endometrial carcinoma is a common malignancy caused by abnormal glandular hyperplasia and companied by aberrant genetic and protein expression [21]. The global death rate of EC has continued to increase due to the lack of precise molecular targets. Though the high-throughput platforms provided promising targets in medical oncology, most previous studies only targeted individual database leading to poor clinical application and differential research bias that lacked biological effects. Our present study integrated RNA expression data from multiple datasets in combination with weighted gene co-expression network analysis to screen out biomarkers, which would provide more reliable and accurate clinical biomarkers for prognostic prediction and molecular therapy of EC.

In this study, unlike a single cohort study, four datasets including GSE17025, GSE63678 and GSE115810 and TCGA UCEC were analyzed systematically for the first time. Liu et al. [22] have just analyzed the dataset GSE17025 and demonstrated that eleven genes were associated with progression and prognosis of endometrial cancer (EC). Although our research is consistent in methodology, we have included as many databases as possible for comprehensive research. Moreover, we screened out the two highly upregulated genes and conducted a series of *in vitro* experiments for functional verification in endometrial cancer. Huo et al. [23] explored the microarray-based dataset GSE72708 by WGCNA and identified six hub genes related to the prognosis of endometrial cancer especially by AKT1 regulation. They just focused on investigating the potential mechanisms of AKT1 in EC, which was complementary to our research results.

After screening DEGs in all datasets, 45 DEGs were obtained. Meanwhile, we applied WGCNA to explore the key modules and core genes correlated with clinical traits of EC by R packages and selected modules that were most definitely associated with the EC disease status. Then after overlapping with DEGs, the most central and connected sixteen hub genes were identified. Next we investigated somatic mutations and copy-number alterations of the top ten genes based on TCGA data, showing that mutation frequency of hub genes were lower than 8%. Furthermore, hub gene-related pathways were analyzed by ClueGO and CluePedia [24], which showed enrichment in mitotic nuclear division, GTPase regulator activity and NADH regeneration pathway, consistent with the results of DAVID and Metascape.

Subsequently, we verified the protein expression of hub genes by GEPIA, UALCAN and HPA databases [25], and then explored their methylation status, which were related with the expression of the genes. Kaplan Meier-plotter was utilized to demonstrate survival analysis and ROC curves were used to assess the sensitivity and specificity of the survival prediction based on gene expression, indicating that they could serve as biomarkers to predict patients’ survival.

Among these core genes, we chose two rarely reported ones which were also dramatically upregulated in EC cells, namely TICRR and PPIF for further studies. Through a series of *in vitro* experiments, we found that silencing TICRR or PPIF resulted in a significant suppression of cell proliferation and migration and elevated the effect of progesterone, suggesting their critical role in the progression of endometrial carcinoma.

To further identify their correlations with infiltrating immune cells, we explored in TISIDB dataset and found that TICRR was associated with activated CD4 T cell and PPIF was correlated with CD56dim natural killer cell, implying that immunocytes may play an essential role in tumor microenvironment. Once the underlying immune-related mechanisms were clarified by experimental method, TICRR and PPIF may be useful for novel immunotherapy. Based on previous studies, TICRR, a crucial checkpoint and replication modulator [26], contributed to the occurrence of tumor through promoting DNA replication and cyclin-dependent kinase regulated the length of S phase by TICRR/TRESLIN phosphorylation in tumor [27], which were consistent with our GSEA and GSVA analysis but had not been validated in related study of endometrial cancer. Additionally, PPIF was reported to be involved in mitochondrial permeability transition regulated necrosis and necroptosis [28, 29], while the correlation between PPIF and mitochondrial ROS production in the development of endometrial carcinoma had not been researched. Besides, we searched DrugBank database to find drugs that targeted oncogenes, showing that cyclosporine and triglyme may be of great value to suppress tumor growth by inhibiting the expression of PPIF, which had not been verified in both experimental and clinical study of endometrial carcinoma.

In conclusion, by combining four databases, WGCNA and multiple bioinformatics methods and *in vitro* experiments, we explored significant gene modules and identified several robust DEGs in EC. Two hub genes (TICRR and PPIF) were robustly overexpressed in EC tissues and the expression profile were closely associated with promoter hypomethylation. GSEA and GSVA further demonstrated that the core genes were highly involved in the development of EC. Further research needs to be implemented to thoroughly elucidate their underlying contribution to the pathogenesis of endometrial cancer and to verify their efficiency as potential prognostic markers and therapeutic targets in endometrial cancer.

## MATERIALS AND METHODS

### Standardization and identification of DEGs

Normalized data of GSE17025, GSE63678 and GSE115810 were obtained from Gene Expression Omnibus (GEO, http://www.ncbi.nlm.nih.gov/geo/) and annotated according to the Affymetrix Human Genome U133A 2.0 Array platform. Pre-processing procedures included RMA background correction, and the “affy” R language package was adopted to complete log2 transformation, quantile normalization and median polish algorithm summarization. Based on the probe annotation information, we mapped the probes to gene symbols. Probes matched with multiple genes were removed and when multiple probes corresponded to one specific gene, the average expression value was considered its final expression. Then, the gene expression profiles were utilized quantile normalization. The TCGA UCEC data was downloaded from TCGA database (https://portal.gdc.cancer.gov/), including 333 endometrial carcinomas and 35 normal tissues. After preprocessing and standardization of data, Limma and DESeq2 R packages were utilized to explore differentially expressed genes (DEGs) [18, 30]. The value of |log2FC| (fold change) >2.00 and P value <0.05 were considered as the cutoff criteria.

### Weighted gene co-expression network construction and identification of modules

We adopted the top 50% most variant genes in each dataset to construct the co-expression network using WGCNA package [31], which was applied to explore clinical traits-related modules and core genes among them. To ensure the reliability of network construction, outlier samples which were defined as connectivity less than -2.5 were removed [32]. Based on the standard scale-free networks, the appropriate soft threshold power was selected and adjacencies were calculated by a power function (scale free R^2^ = 0.85). Then we transformed the adjacency into topological overlap matrix (TOM), and dissimilarity TOM ((1-TOM)) was obtained. Genes with high absolute correlation were divided into gene modules with the dynamic tree cut method by the hierarchical clustering genes using 1-TOM as the distance measure and a deepSplit value of two and a minimum size cutoff of 30 for the generated dendrogram. Highly similar modules were merged with a height cut-off of 0.25. Then we constructed module–trait relationships. The module eigengenes (MEs), the main component of the gene expression profile, represented the entire characteristics of module genes. The correlations between ME and clinical traits were calculated using the Pearson’s correlation analysis [33]. For the intramodular analysis, gene significance (GS) was the absolute value to reflect the relationship between certain expression profile and each trait and the module membership (MM) was utilized to describe the correlation between each ME and gene expression profile. Next, the module membership (MM) and the absolute value of gene significance (GS) were also measured to screen out hub genes. Those with gene significance (GS) > 0.3 and module membership (MM) > 0.8 were identified as hub genes [34].

### Function enrichment analyses of DEGs

To obtain more comprehensive biological information of DEGs, we utilized ClueGO and CluePedia, the plug-in of Cytoscape [35, 36], which can visualize the biological features for genes in a functionally grouped network. Meanwhile, The Database for Annotation, Visualization and Integrated Discovery (DAVID) [37] and Metascape (http://metascape.org/) which offers a biologist-oriented resource for the analysis of systems-level databases [38] were used to verify enriched biological themes pathways. P < 0.05 was defined as the threshold criterion.

### Methylation and gene expression analyses of candidate genes

The somatic mutations and copy number variation of hub genes were explored in TCGA database by cBioPortal [39], which enables us to interactively investigate genetic alterations across samples and genes. Then Gene Expression Profiling Analysis (GEPIA, http://gepia.cancer-pku.cn/) was used to demonstrate the gene expression profiles from TCGA database. MEXPRESS and UALCAN analysis was performed to detect DNA methylation status [16]. The immunohistochemistry (IHC) data of common genes was searched in Human protein atlas (HPA) project which contains detailed information of samples [40].

### Survival analysis of hub genes

We performed survival analysis of genes by log-rank tests and Kaplan–Meier plotter (http://kmplot.com/analysis/) to explore differences in survival between different groups [41]. Besides, in order to evaluate hub genes’ predictive values, we drew receiver operating characteristic (ROC) curves and measured area under the ROC curve (AUC) and 95% confidence intervals (CI) with SPSS software [42].

### Correlation between gene expression and tumor-infiltrating immune cells

To explore the association between the expression of identified hub genes and infiltrating immune cells, such as lymphocytes, immunomodulators and chemokines, we utilized the online tool TISIDB (http://cis.hku.hk/TISIDB/index.php) [43], which integrates multiple heterogeneous data types for each gene based on TCGA database.

### Data processing of gene set enrichment analysis (GSEA) and gene set variation analysis (GSVA)

We separated endometrial cancer cases from TCGA UCEC data into high-risk and low-risk groups by using the optimal cut-off values of selected hub gene. To identify potential biological pathways of the hub genes, GSEA software 4.0.3 (https://software.broadinstitute.org/gsea/index.jsp) was implemented in this study [44]. Similarly, the “GSVA” package in R language software 3.6.3 was utilized to explore the pathways most associated with core genes [45]. The gene sets “c2.cp.v7.2.symbols.gmt” and “c5.go.v7.2.symbols.gmt” that were downloaded from the Molecular Signature Database (MSigDB, http://software.broadinstitute.org/gsea/msigdb/index.jsp) were regarded as the reference gene set. Terms with FDR less than 0.25 and P value less than 0.01 were regarded as statistically significant pathways.

### Cell lines and transient transfection

The human endometrial epithelial cells (EEC) were purchased from American Type Culture Collection (ATCC, VA, USA) and were cultured in Epithelial Cell Medium (MingZhoubioCO.,Ltd). The human endometrial cancer cell lines (Ishikawa and HEC-1-B) were conserved in our team and were cultured in DMEM/F12 medium with 10% fetal bovine serum (FBS, Gibco). Cells were transfected using Lipofectamine 3000 (Invitrogen, NY, USA) according to the manufacturer’s protocol. The siRNAs for TICRR and PPIF were designed by GenePharma (Shanghai, China) and the sequences were listed in Table 3.

**Table 3:** The sequences for siRNAs.

**Gene**			**Sequences (5’-3’)**
TICRR	siTICRR-1	Sense:	5’-CCAACUGAUGCCACUUUAATT-3’
		Antisense:	5’-UUAAAGUGGCAUCAGUUGGTT-3’
	siTICRR-2	Sense:	5’-CCGAGACUCCAGUGCAUAATT-3’
		Antisense:	5’-UUAUGCACUGGAGUCUCGGTT-3’
PPIF	siPPIF-1	Sense:	5’-GGGUGAUCCCUUCCUUCAUTT-3’
		Antisense:	5’-AUGAAGGAAGGGAUCACCCTT-3’
	siPPIF-2	Sense:	5’-GCUAAUGCUGGUCCUAACATT-3’
		Antisense:	5’-UGUUAGGACCAGCAUUAGCTT-3’

### Real-time PCR analysis

Cells were seeded into 6-well plates. After the experimental treatment, total RNA was extracted with Trizol reagent and was reverse transcribed into cDNA. Then Real time PCR was performed using SYBR Premix (Takara, China). Primers were listed in Table 4.

**Table 4:** Sequences of primers used for amplification of target genes.

**Gene**	**primer nucleotide sequence**
UBE2C	Forward: 5’-GCAGTCGTGTTCTCCGAGTT-3’
	Reverse: 5’-GCTCCTGCTGTAGCCTTTTG-3’
LYPLA2	Forward: 5’-AAGAAGGCAGCAGAGAACATC-3’
	Reverse: 5’-CTCCCAGGACGATTCGATTG-3’
PPIF	Forward: 5’- TGGTGACACAGGCCACAGAC-3’
	Reverse: 5’- CCGGAGCACAGGAGCTTACA-3’
BIRC5	Forward: 5’-GACGACCCCATGCAAAGGAA-3’
	Reverse: 5’- GTGGCACCAGGGAATAAACC-3’
ABT1	Forward: 5’-ACGGGTAGTGCCAGGTATTG-3’
	Reverse: 5’-CGGTCCTCAGCCTGAAAGAA-3’
PRRC2A	Forward: 5’-GCCACAGGGATTCCCAATCA-3’
	Reverse: 5’-TTGGGGGAGTTGCCCTTTTT-3’
TICRR	Forward: 5’- CACGGGAGACGAAGAGGT-3’
	Reverse: 5’- CTGGAACAGCAGCGGAGA-3’
ASCC1	Forward: 5’-CTCACCACGACGAGGACCG-3’
	Reverse: 5’-TCCAATTATGCCCGTGAGGG-3’
WT1	Forward: 5’-CCAAATGACATCCCAGCTTG-3’
	Reverse: 5’-GTGTGGTTATCGCTCTCGTAC-3’
SOWAHC	Forward: 5’-CTGGTCAAGCGGGACTTCAT-3’
	Reverse: 5’-CTCCGACTCAGGTACTGGGA-3’
E-cadherin	Forward: 5′-CGAGAGCTACACGTTCACGG-3′
	Reverse: 5′-GGGTGTCGAGGGAAAAATAGG-3′
N-cadherin	Forward: 5′-TGCGGTACAGTGTAACTGGG-3′
	Reverse: 5′-GAAACCGGGCTATCTGCTCG-3′
Vimentin	Forward: 5′-TGCCGTTGAAGCTGCTAACTA-3′
	Reverse: 5′-CCAGAGGGAGTGAATCCAGATTA-3′
Snail	Forward: 5′-ACTGCAACAAGGAATACCTCAG-3′
	Reverse: 5′-GCACTGGTACTTCTTGACATCTG-3′
Twist	Forward: 5′-ATTCAAAGAAACAGGGCGTGG-3′
	Reverse: 5′-CCTTTCAGTGGCTGATTGGC-3′
MMP9	Forward: 5′-TTGACAGCGACAAGAAGTGG-3′
	Reverse: 5′-GCCATTCACGTCGTCCTTAT-3′
MMP2	Forward: 5′-TCTCCTGACATTGACCTTGGC-3′
	Reverse: 5′-CAAGGTGCTGGCTGAGTAGATC-3′
CCND1	Forward:5’-AAACAGATCATCCGCAAACAC-3’
	Reverse:5’-GTTGGGGCTCCTCAGGTTC-3’
MYC	Forward:5’-CCTGGTGCTCCATGAGGAGA-3’
	Reverse:5’-TCCAGCAGAAGGTGATCCAGAC-3’
GAPDH	Forward: 5’-ACCCAGAAGACTGTGGATGG-3’
	Reverse: 5’-TCAGCTCAGGGATGACCTTG-3’

### Western blotting (WB) assay

Total protein was extracted using radioimmunoprecipitation assay buffer (Beyotime, China) with inhibitor cocktail, and the concentrations were quantified by bicinchoninic acid assay kit (Yeasen, China). Western blotting was done according to standard protocols. Antibodies against TICRR (1:1000, NBP2-41283), PPIF (1:1000, 18466-1-AP), β-tubulin (1:5000, 30303ES10), GAPDH (1:5000, 30203ES10) were used as primary antibodies. The immunoblot bands intensity were quantified with ImageJ software (NIH, MD, USA).

### Immunohistochemical (IHC) staining

Paraffin sections of twelve pairs of endometrial adenocarcinoma (EC) and the paracancer tissue from the same patient were obtained from the tissue bank at the International Peace Maternity and Child Health Hospital. This study was in accordance with the tenets of the Helsinki Declaration. Immunohistochemical staining was performed as previously described [7]. Primary antibodies used in IHC assay included TICRR (NBP2-41283, Novus), PPIF (18466-1-AP, Proteintech) and a rabbit immunoglobulin G was utilized as a negative control. All sections were dual scored by two experienced pathologists who were blinded to sample information based on the combination of the intensity score and the percentage score.

### Flow cytometric analysis

The percentage of apoptosis cells were detected by flow cytometry using the Annexin V-FITC staining kit (BD Pharmingen, USA). Briefly, after transfected with siRNA for 24 h, cells were digested, collected and washed three times with PBS, and then resuspended in binding reagent. Annexin v-FITC and PI were then added according to the manufacturer's instructions. A FACS can flow cytometer and FlowJo software (Tree Star Inc., OR, USA) were utilized to analyze the data.

### Cell proliferation and plate clonality assays

The proliferation assay was assessed using Cell Counting Kit-8 (CCK-8). Briefly, cells were seeded into 96-well plates (2 × 10^3^ cells/well). After transfection and drug treatment, the absorbance was measured at different points of time by a microplate reader (Bio-Rad, CA, USA). For colony formation assays, 500 cells were seeded in 6-well plates and cultured for two weeks. After fixation and staining, colony quantity was counted and photographed under the optical microscope.

### Cell migration and invasion assays

For cell migration assay, transfected Hec-1B cells (5× 104) were suspended in 200 μL of serum-free medium and seeded onto the upper chambers of the 24-well plates with 8-μm-pore filters (Corning, NY, USA). 20% fetal bovine serum was added to the lower chamber medium. For cell invasion assay, the membranes of the top chambers were coated with Matrigel (BD, USA). After using drugs for 18-24h, the crossed cells were fixed, stained and counted at 100×magnifcation.

### Cell wound-healing assay

When cells reached 90% confluence in 6-well plates, a wound was scratched by utilizing with a 10-μL micropipette tip. After washing with PBS for three times, the cells were incubated in serum-free media for 48h. The images were photographed under the inverted microscope.

### Statistical analysis

All experiments were performed at least three times. R language software 3.6.3 was used in this study and the data presentation was conducted using Graphpad Prism 7. Statistical analyses between groups were assessed using the Student's t-test. Data are shown as mean ± SD. P-values < 0.05 were defined as statistically significant.

### Data availability statement

The authors confirm that all data analyzed in the current study are available within the paper and its supplementary files.

## Supplementary Material

Supplementary Figures

Supplementary Table 1

Supplementary Table 2

Supplementary Table 3
