# Supplementary material for: Overexpression of TICRR and PPIF confer poor prognosis in endometrial cancer identified by gene co-expression network analysis
Source: Aging (Albany NY). 2021 Jan 20;13(3):4564–89. doi: 10.18632/aging.202417 (PMC7906164; doi:10.18632/aging.202417)
Supplement: Supplementary Figures [file aging-13-202417-s001.pdf]

SUPPLEMENTARY FIGURES

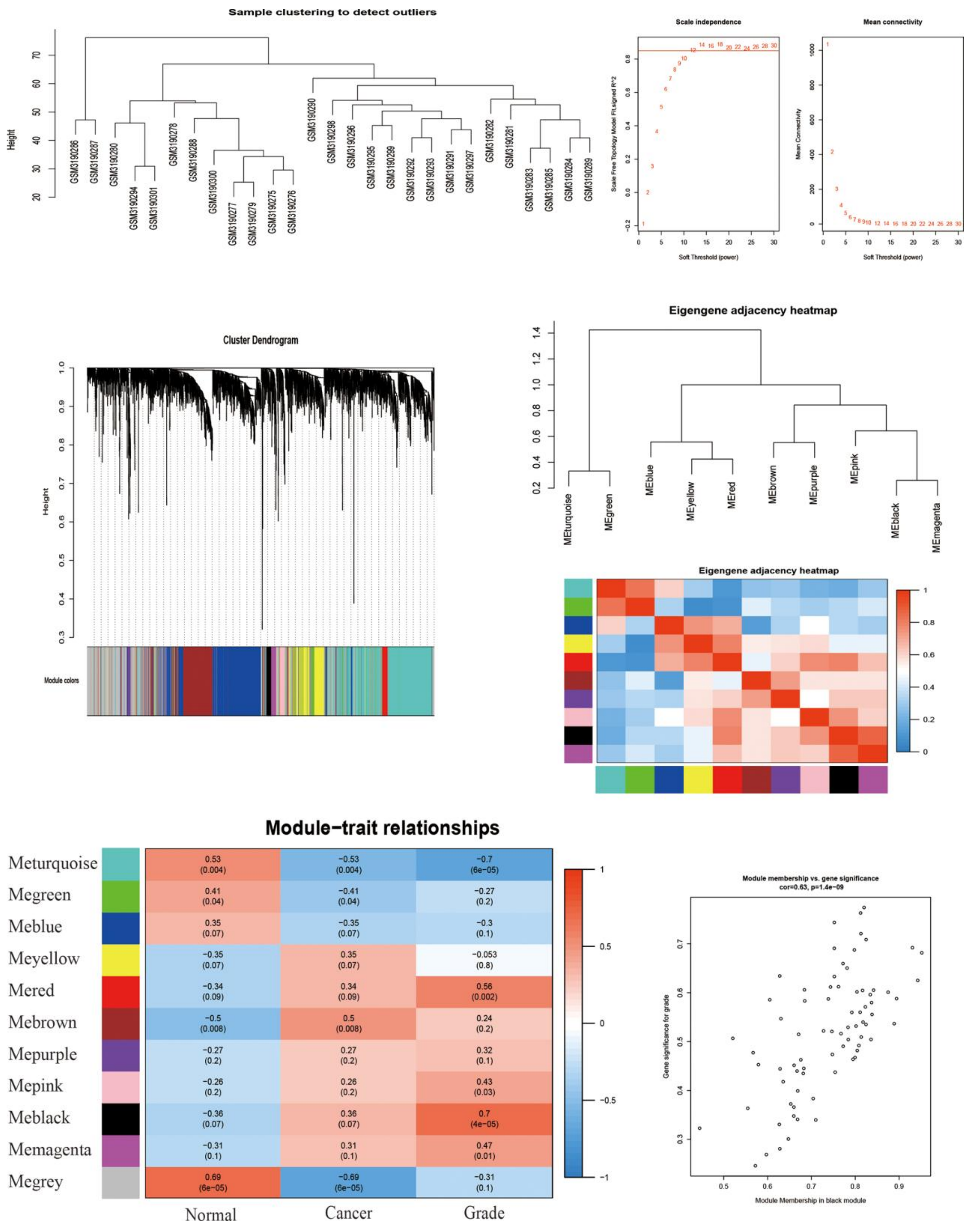

Supplementary Figure 1. Co-expression network analysis for GSE115810.

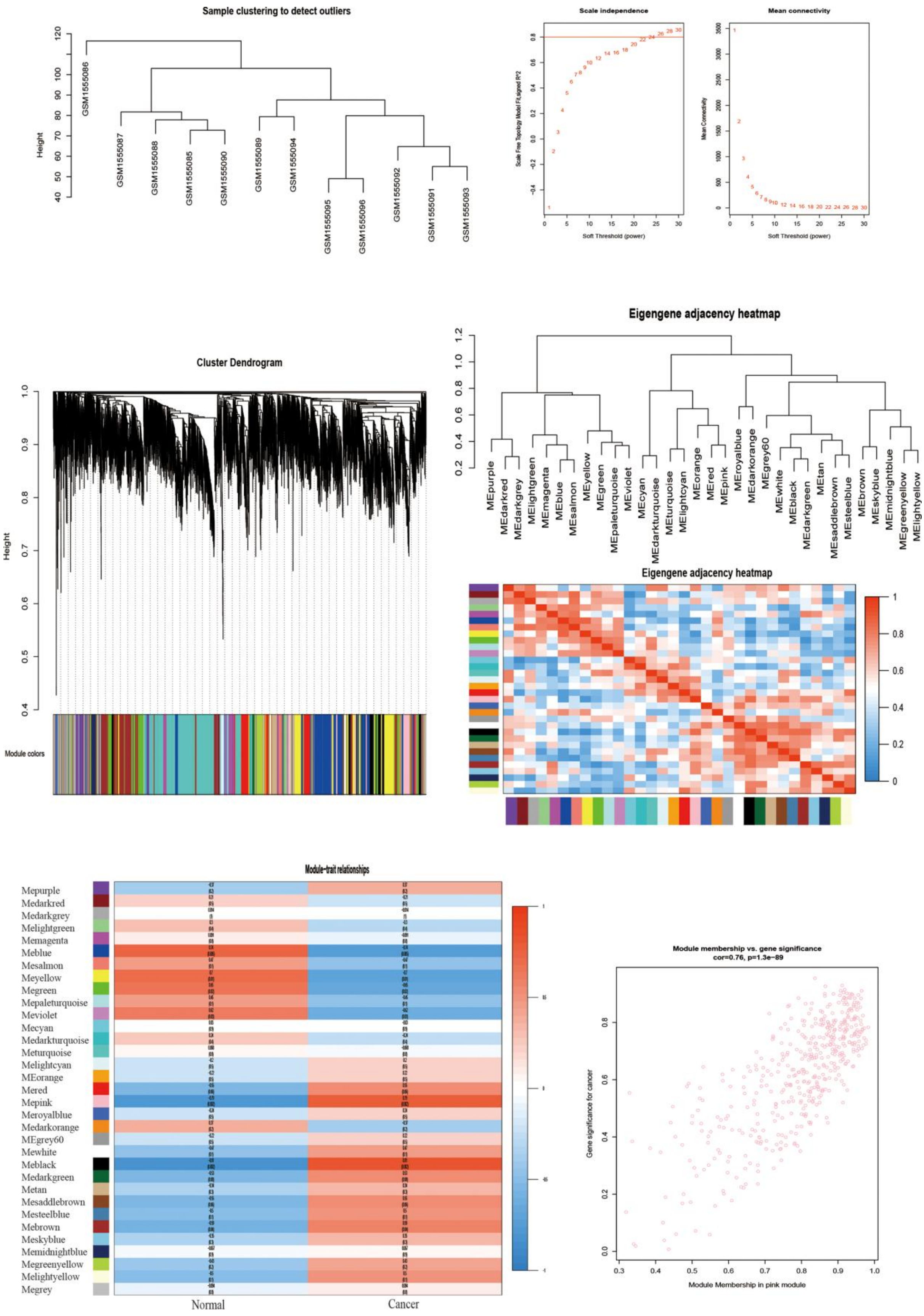

Supplementary Figure 2. Co-expression network analysis for GSE63678.

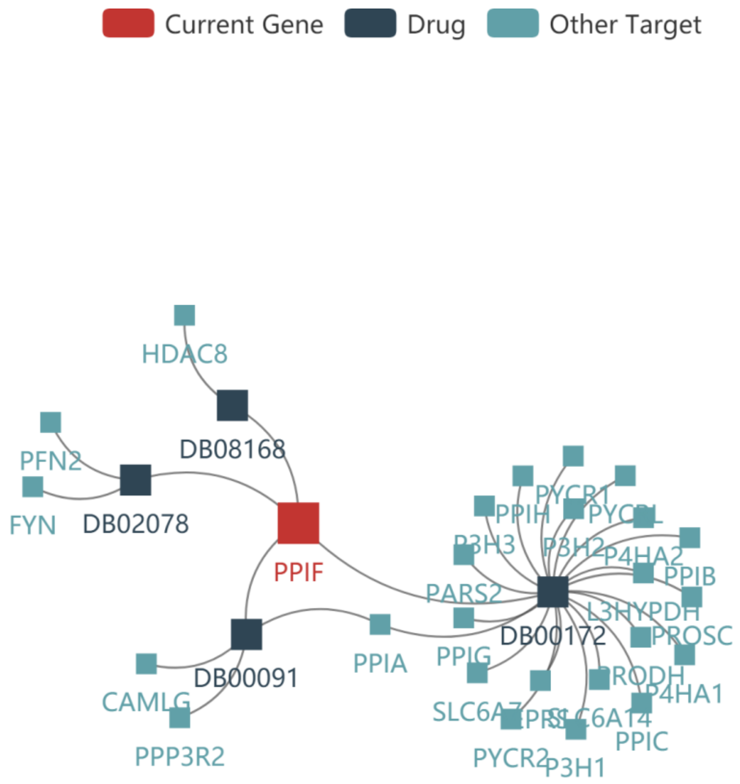

Supplementary Figure 3. Drugs targeting PPIF collected from DrugBank database. DB00091: Cyclosporine, DB00172: L-Proline, DB02078: Triglyme, DB08168: 7-AMINO-4-METHYL-CHROMEN-2-ONE.
