# Supplementary material for: Overexpression of TICRR and PPIF confer poor prognosis in endometrial cancer identified by gene co-expression network analysis
Source: Aging (Albany NY). 2021 Jan 20;13(3):4564–89. doi: 10.18632/aging.202417 (PMC7906164; doi:10.18632/aging.202417)
Supplement: Supplementary Table 3 [file aging-13-202417-s004.pdf]

Supplementary Table 3. Detailed lists of the 16 common genes.

DEGs	WGCNA	DEGs|WGCNA
HSPA4	HMGB1P1	UBE2C
ABLIM1	ABCF1	SOWAHC
PRKRIRP6	ABCF2	CYR61
DDHD1	ABCG1	MYO15B
SLC18A1	ACAA2	ARHGAP10
STYX	ACAT2	GNG11
ANP32B	ACBD7	LYPLA2
BCAS2P2	ACE	PLS1
C17orf98	ACOT7	PPIF
UGT1A6	ACP1	BIRC5
ROPN1L	ACVR2B	PRRC2A
SMIM5	ADD2	TICRR
C20orf202	ADIPOR2	MUC1
RN7SL801P	ADSL	VAMP8
RN7SL567P	AFG3L2	WT1
ATAD2B	TROAP	CXCL12
PEAR1	LINC00667	
RNA5SP18	NDN	
HMGN1P3		
BET1		
C14orf159		
CLEC10A		
STAMBPL1		
SDHCP3		
PCED1A		
PNMA1		
CSNK1A1P1		
RN7SL446P		
SLC25A36		
BNIP3P30		
YIPF7		
SORT1		
MYO9A		
ARMC8P1		
CMTR1		
CEACAM8		
ADAMTS16		
ASCC1		
ABT1		
